# Association between carotid intima-media thickness and index of central fat distribution in middle-aged and elderly Chinese

**DOI:** 10.1186/s12933-014-0139-2

**Published:** 2014-10-30

**Authors:** Chenxi Ren, Jie Zhang, Yu Xu, Baihui Xu, Wanwan Sun, Jichao Sun, Tiange Wang, Min Xu, Jieli Lu, Weiqing Wang, Yufang Bi, Yuhong Chen

**Affiliations:** Department of Endocrinology and Metabolism, Shanghai Clinical Center for Endocrine and Metabolic Diseases, Shanghai Institute of Endocrine and Metabolic Diseases, Rui-Jin Hospital, Shanghai Jiao-Tong University School of Medicine, 197 Rui-Jin 2nd Road, Shanghai, 200025 China; Key Laboratory for Endocrine and Metabolic Diseases of Ministry of Health, Rui-Jin Hospital, Shanghai Jiao-Tong University School of Medicine, E-Institute of Shanghai Universities, Shanghai, 200025 China; Institute of Health Sciences, Shanghai Institutes for Biological Sciences, Chinese Academy of Sciences and Shanghai Jiao-Tong University School of Medicine, Shanghai, 200031 China

**Keywords:** Waist-to-height ratio, Visceral fat area, Carotid intima-media thickness

## Abstract

**Background:**

Evidence has demonstrated that central fat distribution produces the most profound metabolic abnormalities and is associated with an increased risk of atherosclerotic cardiovascular diseases. We aimed to investigate whether the indexes of central fat distribution, including waist-to-height ratio (WHtR) and visceral fat area (VFA), were stronger risk factors of subclinical atherosclerosis than body mass index (BMI) in Chinese adults.

**Methods:**

A total of 3381 participants aged 40 years or older without history of cardiovascular diseases (CVD) were enrolled in the present cross-sectional study from the Songnan community, Shanghai, China. Height, weight and waist circumference (WC) were measured by experienced physicians. High-resolution B-mode ultrasonography was performed to measure carotid intima-media thickness (CIMT). Regional adiposity was measured by a dual-source computed tomography (CT) scanner.

**Results:**

Normal weight but central obesity group (BMI < 23 kg/m^2^ and WHtR > 0.5) had higher levels of systolic blood pressure (SBP), fasting plasma glucose (FPG), 2 h post-load glucose (2 h PG), Hemoglobin A1c (HbA1c), and CIMT, as well as an elevated prevalence of hypertension and diabetes compared with overweight/obesity but not central obesity group (BMI ≥ 23 kg/m^2^ and WHtR ≤ 0.5). In logistic regression analysis, WHtR > 0.5 was significantly and independently associated with elevated CIMT (odds ratio [OR] 1.30, 95% confidence interval [CI] 1.01-1.68, p value = 0.044). Similar association was noted for each standard deviation (SD) increase of WHtR (OR 1.25, 95% CI 1.07-1.47, p value = 0.006). Stepwise multiple linear regression analysis revealed that both WHtR and VFA were important determinants of CIMT, independent of other well-recognized risk factors (both p values < 0.01).

**Conclusions:**

WHtR and VFA were associated with CIMT, independent of BMI and conventional CVD risk factors. Given the relatively high cost and complexness of VFA measurement, WHtR could be a more convenient and appropriate measure of abdominal obesity in clinical practice.

## Background

As known to all, overweight and obesity are international problems and associated with metabolic disorders and cardiovascular diseases (CVD) [1,2]. In recent years, evidence has demonstrated that abdominal accumulation of fat tissue produces the most profound metabolic abnormalities and is associated with an increased risk of atherosclerotic CVD [3]. Accumulation of visceral fat is a major component of central obesity which is more important for the development of metabolic disorders including insulin resistance [4-6], type 2 diabetes [4,7,8], and metabolic syndrome [9]. Anthropometric measurement is useful in clinical practice due to its convenience and non-invasiveness [10]. The waist-to-height ratio (WHtR) is an important anthropometric index of central obesity that circumvents the limitations of waist circumference (WC) [11]. Because of the inclusion of height into the index, any potential confounding of cardiometabolic risk by height is avoided. A prospective study [12] showed that several different measures of abdominal obesity [WHtR > WC > waist-to-hip ratio (WHR)] were strong predictors of stroke among more than 45,000 women below age 60. WHtR is also a better predictor than WC for diabetes, dyslipidaemia, hypertension and CVD risk in both sexes in populations of various nationalities and ethnic groups [12]. In addition, there are also more complex measurements on central adiposity that could improve CVD prediction above and beyond what have already been achieved by simple anthropometric measures [13]. Among them, visceral fat area (VFA) is an accurate and important indicator of central fat distribution.

Carotid intima-media thickness (CIMT) is accepted as an indicator of subclinical atherosclerosis [14] and a risk factor for myocardial infarction and stroke in elderly adults [15]. However, few studies have investigated the association of VFA with CIMT in Chinese population. Furthermore, little evidence is available on the association between WHtR and subclinical atherosclerosis in various body mass index (BMI) categories. In the current study, we aimed to investigate the association between CIMT and indexes of central fat distribution (WHtR and VFA) in a middle-aged and elderly Chinese population.

## Methods

### Study sample

A total of 4012 subjects were recruited from the Songnan community, Baoshan, Shanghai in 2009. All the residents aged 40 years old or above were invited to receive measurements of weight, height and WC. WHtR was calculated as WC divided by height in the same unit. BMI was calculated as weight in kilograms divided by height in meters squared. Based on the calculation of WHtR and BMI, participants were categorized into four groups: 1) normal group: BMI < 23 kg/m^2^ and WHtR ≤ 0.5; 2) overweight/obesity but not central obesity group (OW/OB-NC): BMI ≥ 23 kg/m^2^ and WHtR ≤ 0.5; 3) normal weight but central obesity group (NW-C): BMI < 23 kg/m^2^ and WHtR > 0.5; and 4) overweight/obesity and central obesity group (OW/OB-C): BMI ≥ 23 kg/m^2^ and WHtR > 0.5. We excluded those people who had a history of previous cardiovascular diseases (myocardial infarction, unstable angina, percutaneous coronary intervention, or stroke), or impaired liver or renal function (aminotransferase more than twice the upper limit of the normal range, serum creatinine level greater than 133 μmol/l [1.5 mg/dl] or estimated glomerular filtration rate less than 60 ml/min/1.73 m^2^), who were pregnant or having significant medical comorbidities, or had missing data on CIMT and WHtR. Finally, 3381 participants were included in analysis. In addition, a subgroup of participants underwent coronary computed tomography (CT) angiography to detect coronary atherosclerosis and abdominal CT scanning to measure subcutaneous and visceral fat. The subgroup included all participants with diabetes or prediabetes, and 50% of participants with normal glucose regulation using simple random sampling, from the original 4012 participants after excluding those with following characteristics: 1) age over 60, 2) having symptoms of coronary artery disease, 3) having a history of previous CVD, 4) having abnormal Q waves on resting electrocardiogram (ECG), 5) with a prior diagnosis of diabetes more than 5 years, 6) having impaired liver or renal function, 7) being pregnant or having significant medical comorbidities, 8) having X-ray examination or CT scan within one year, 9) with tachycardia or arrhythmia such as atrial fibrillation on ECG, and 10) having a history of allergic reaction to iodine-containing contrast agent. A total of 942 participants were recruited and 593 responded, among which 569 had abdominal CT scanning. After excluding participants with missing data on CIMT, blood pressure (BP), and 2 h post-load glucose (2 h PG), 542 participants were eventually included for the subgroup analysis.

This study was approved by the Institutional Review Board of Rui-Jin Hospital, Shanghai Jiao-Tong University School of Medicine and was in accordance with the principle of the Helsinki Declaration II. The written informed consent was obtained from each participant.

### Measurement of CIMT

CIMT on the far wall of the right and left common carotid arteries, 1.5 cm proximal to the bifurcation, was measured by a trained sonographer using a high-resolution B-mode tomographic ultrasound system (Esaote Biomedica SpA, Genova, Italy) with a linear 7.5-MHz transducer. The transducer was manipulated so that the lumen diameter was maximized in the longitudinal plane. CIMT was measured online during end diastole as the distance from the leading edge of the first echogenic line to that of the second echogenic line. The first and second lines represent the lumen-intimal interface and the collagen-contained upper layer of tunic adventitia, respectively. The greater value of the right and left common CIMT was used for analysis.

### Measurement of VFA

**Figure 1:**
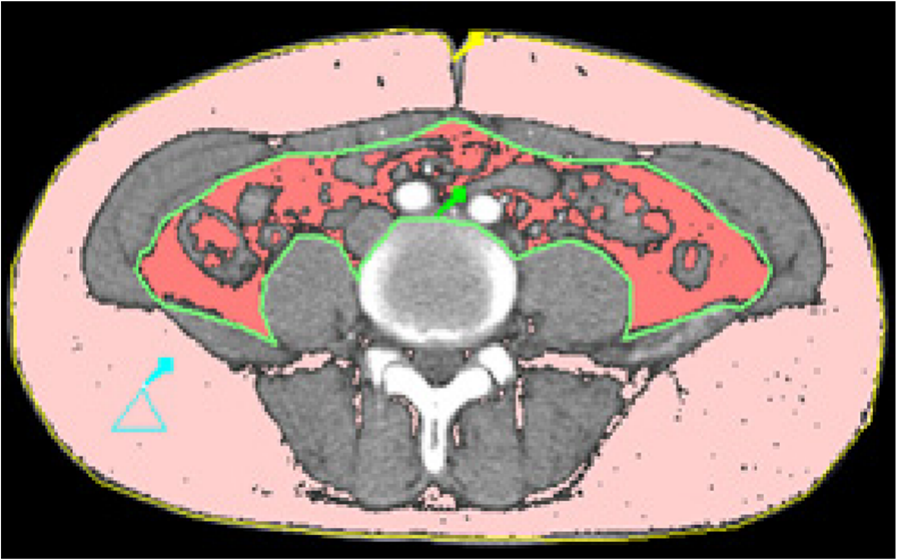
**Distribution of abdominal fat as measured by FatScan software on a CT image.** The visceral fat was indicated in red, and the subcutaneous fat was indicated in pink.

Regional adiposity was measured by a dual-source CT scanner (SOMATOM Definition, Siemens Medical Solutions, Forchheim, Germany). Visceral and subcutaneous adiposity were measured at the midpoint between the fourth and fifth lumbar vertebrae with participants in the supine position. Fat area was calculated by FatScan version 2.0 software (N2 System Co., Osaka, Japan) by two independent observers. The intraperitoneal area was defined manually by tracing its contour (the green line) and the outline of abdominal wall was also circled (the yellow line) on CT images using the software, which calculated VFA and subcutaneous fat area (SFA) automatically (Figure 1). If readings between two observers differed by more than 10%, a third observer who did not know the results reanalyzed the images.

### Assessment of covariates

The detailed information about medical history and lifestyle including smoking and drinking status was obtained using a standard questionnaire by trained physicians. Current smoking status was defined as yes if the subject smoked at least one cigarette per day or seven cigarettes per week in the past 6 months. Current drinking status was defined as yes if the subject consumed alcohol at least once a week in the past 6 months. Body height, weight, and WC were measured by experienced physicians. Height and weight were recorded to the nearest 0.1 cm and 0.1 kg while participants were wearing light indoor clothing without shoes. WC was measured to the nearest 0.1 cm with participants in the standing position. BP was measured at the non-dominant arm in a seated position after a 5-min rest using an automated electronic device (OMRON Model HEM-752 FUZZY’ Omron Co., Dalian, China). Three measurements were taken in one minute apart and the average of the three measurements was used in analysis.

All participants received a two-point (at 0 and 2 hrs) 75-g oral glucose tolerance test after an overnight fast of more than 10 hrs. The measurements of fasting plasma glucose (FPG), 2 h PG, triglycerides (TG), total cholesterol (TC), low-density lipoprotein cholesterol (LDL-C), and high-density lipoprotein cholesterol (HDL-C) were performed on an autoanalyzer (ADVIA-1650 Chemistry Analyzer; Bayer Diagnostics, Tarrytown, NY, USA). Hemoglobin A1c (HbA1c) was measured by high performance liquid chromatography (HPLC; Bio-Rad, Hercules, CA, USA). The index of homeostasis model assessment of insulin resistance (HOMA-IR) was calculated according to the formula: HOMA-IR = fasting insulin concentrations (mIU/L) × FPG concentrations (mmol/L)/22.5.

### Definitions

Central obesity was defined as WHtR > 0.5 for males and females [16].

Overweight/obesity was defined as BMI ≥ 23 kg/m^2^ in Asians [17,18].

### Statistical analysis

SAS version 9.1 (SAS Institute, Cary, NC) was used for database management and statistical analysis. Data are presented as means ± SD or medians (interquartile ranges) for continuous variables or numbers (percentages) for categorical variables. Measurements with a skewed distribution, such as TG, HbA1c, HOMA-IR, and CIMT were normalized by logarithmic transformation. Comparisons of means and proportions were performed with variance analysis and x^2^ tests, respectively. Pearson correlation coefficients were used to measure linear associations between different risk factors and CIMT. Stepwise multiple linear regression analysis was used to identify independent determinants of CIMT. In addition, we used the logistic regression analysis to evaluate the association of WHtR with elevated CIMT by categorized WHtR (WHtR ≤ 0.5 and WHtR > 0.5) using WHtR ≤ 0.5 as the reference as well as per standard deviation (SD) increment of WHtR. In the present study, subjects with CIMT values above the upper quartile (≥ 0.7 mm) of the entire study population were defined as those with elevated CIMT. Adjusted odds ratio (OR) and corresponding 95% confidence interval (CI) were calculated and a p value < 0.05 was considered statistically significant.

## Results

### Clinical characteristics of the study population

Compared with the normal group, not only OW/OB-C but also NW-C group had higher systolic BP (SBP), diastolic BP (DBP), FPG, 2 h PG, HbA1c, HOMA-IR, TC, TG, LDL-C and CIMT, and lower HDL-C, as well as an elevated prevalence of hypertension and diabetes. Compared with OW/OB-NC group, NW-C and OW/OB-C groups had higher SBP, FPG, 2 h PG, HbA1c, CIMT, and the prevalence of hypertension and diabetes. There was no significant difference of lipid profiles between OW/OB-NC and NW-C groups (p value > 0.05). However, TC, TG, and LDL-C gradually increased and HDL-C gradually decreased among four groups (p for trend < 0.0001, Table 1).

**Table 1:** **Clinical characteristics of the study population according to WHtR and BMI categories**

	**WHtR ≤ 0.5 (N = 785)**		**WHtR > 0.5 (N = 2596)**		
	**BMI < 23 kg/m** ^**2**^ **(N = 592)**	**BMI ≥ 23 kg/m** ^**2**^ **(N = 193)**	**BMI < 23 kg/m** ^**2**^ **(N = 308)**	**BMI ≥ 23 kg/m** ^**2**^ **(N = 2288)**	**p for trend**
Age (years)	57.0 ± 8.7	54.6 ± 7.2^†^	62.6 ± 10.8^†‡^	60.5 ± 9.5^†‡#^	< 0.0001
Male, n (%)	249 (42.1)	87 (45.1)	97 (31.5) ^†^	929 (40.6) ^‡#^	0.4886
Current smokers, n (%)	143 (24.2)	52 (27.0)	64 (20.8)	468 (20.5)	0.0182
Alcohol drinking, n (%)	102 (17.2)	35 (18.1)	49 (15.9)	433 (19.0)	0.2999
Hypertension, n (%)	179 (30.2)	69 (35.8)	160 (52.0) ^†‡^	1428 (62.4)^†‡#^	< 0.0001
SBP (mmHg)	126.5 ± 19.1	128.9 ± 17.4	138.1 ± 23.3^†‡^	141.1 ± 21.3^†‡#^	< 0.0001
DBP (mmHg)	74.2 ± 9.6	77.8 ± 9.6^†^	77.3 ± 10.2^†^	80.0 ± 10.0^†‡#^	< 0.0001
HbA1c (%)*	6.0 (5.7-6.3)	5.9 (5.6-6.2)	6.0 (5.7-6.6)^†‡^	6.2 (5.8-6.9)^†‡^	< 0.0001
FPG (mmol/L)	5.46 ± 1.88	5.30 ± 1.49^†^	5.80 ± 2.18^†‡^	5.91 ± 1.94^†‡^	< 0.0001
2 h PG (mmol/L)	8.4 ± 5.3	7.6 ± 3.8	9.8 ± 6.0^†‡^	10.4 ± 5.4^†‡^	< 0.0001
HOMA-IR*	0.91(0.61-1.41)	1.26 (0.85-1.80)^†^	1.31 (0.83-2.00)^†^	2.07 (1.38-3.22)^†‡#^	< 0.0001
Diabetes, n (%)	122 (20.6)	27 (14.0)^†^	83 (27.0)^†‡^	796 (34.8)^†‡#^	< 0.0001
TC (mmol/L)	4.98 ± 0.90	5.04 ± 0.88	5.16 ± 0.98^†^	5.21 ± 1.00^†‡^	< 0.0001
TG (mmol/L)*	1.01 (0.77-1.37)	1.19 (0.84-1.84)^†^	1.30 (0.97-1.99)^†^	1.59 (1.14-2.29) ^†‡#^	< 0.0001
LDL-C (mmol/L)	2.19 ± 0.61	2.29 ± 0.62	2.34 ± 0.66^†^	2.45 ± 0.71^†‡#^	< 0.0001
HDL-C(mmol/L)	1.51 ± 0.35	1.38 ± 0.34^†^	1.41 ± 0.33^†^	1.31 ± 0.28^†‡#^	< 0.0001
Anthropometric index					
WHtR	0.46 ± 0.03	0.48 ± 0.01^†^	0.53 ± 0.03^†‡^	0.57 ± 0.05^†‡#^	< 0.0001
BMI (kg/m^2^)	20.6 ± 1.6	24.2 ± 1.1^†^	21.8 ± 1.2^†‡^	26.9 ± 3.0^†‡#^	—
CIMT (mm)*	0.6 (0.5-0.6)	0.6 (0.5-0.6)	0.6 (0.6-0.7) ^†‡^	0.6 (0.6-0.7)^†‡^	< 0.0001

SBP, systolic blood pressure; DBP, diastolic blood pressure; FPG, fasting plasma glucose; 2 h PG, 2 h post-load glucose; HOMA-IR, homeostasis model assessment of insulin resistance; TC, total cholesterol; TG, triglyceride; LDL-C, low-density lipoprotein cholesterol; HDL-C, high-density lipoprotein cholesterol; WHtR, waist-height ratio; BMI, body mass index; CIMT, carotid intima-media thickness.

*Medians with interquartile ranges.

^†^p < 0.05 versus the WHtR ≤ 0.5 and BMI < 23 kg/m^2^ group.

^‡^p < 0.05 versus the WHtR ≤ 0.5 and BMI ≥ 23 kg/m^2^ group.

^#^p < 0.05 versus the WHtR > 0.5 and BMI < 23 kg/m^2^ group.

Compared with NW-C group, OW/OB-C group also had higher SBP, DBP, HOMA-IR, TG, LDL-C, CIMT and lower HDL-C, as well as a higher prevalence of hypertension and diabetes.

### WHtR and CIMT

Simple regression analyses revealed that age, sex, SBP, DBP, FPG, 2 h PG, HbA1c, HOMA-IR, TC, TG, LDL-C, HDL-C, WHtR and BMI were significantly correlated with CIMT (p value < 0.01). After performing multiple stepwise linear regression analysis, we found that age, sex, SBP, DBP, HbA1c, LDL-C and WHtR were independent determinants of CIMT (p value < 0.0001). However, BMI did not enter the final step in stepwise multiple linear regression analysis (Table 2).

**Table 2:** **Univariate and multiple stepwise linear regression analysis of risk factors associated with CIMT**

	**Univariate**		**Stepwise**	
	**r**	**p value**	**β ± SE**	**p value**
Age (years)	0.3848	< 0.0001	0.0039 ± 0.0003	< 0.0001
Sex (male = 1, female = 2)	−0.2379	< 0.0001	−0.0594 ± 0.0062	< 0.0001
SBP (mmHg)	0.3079	< 0.0001	0.0015 ± 0.0002	< 0.0001
DBP (mmHg)	0.0732	< 0.0001	−0.0014 ± 0.0003	< 0.0001
HbA1c (%)	0.2083	< 0.0001	0.1774 ± 0.0317	< 0.0001
FPG (mmol/L)	0.1472	< 0.0001	—	—
2 h PG (mmol/L)	0.1977	< 0.0001	—	—
HOMA-IR	0.1216	< 0.0001	—	—
TC (mmol/L)	0.0394	0.0220	—	—
TG (mmol/L)	0.0485	0.0047	—	—
LDL-C (mmol/L)	0.1148	< 0.0001	0.0220 ± 0.0035	< 0.0001
HDL-C (mmol/L)	−0.0974	< 0.0001	—	—
WHtR	0.1974	< 0.0001	0.1810 ± 0.0418	< 0.0001
BMI (kg/m^2^)	0.1051	< 0.0001	—	—

r, correlation coefficient; β, regression coefficient; SE, standard error.

In logistic regression analysis (Table 3), the risk of elevated CIMT was increased in WHtR > 0.5 group (OR 1.90, 95% CI 1.55-2.35) after adjusted for age, sex, smoking and drinking status (Model 1). WHtR > 0.5 was still significantly associated with elevated CIMT (OR 1.45, 95% CI 1.14-1.83) after further adjustment for SBP, DBP, 2 h PG, HbA1c, HOMA-IR, TG, LDL-C, HDL-C and medical treatment for diabetes and hypertension based on model 1 (Model 2). After adjustment for BMI based on model 2 (Model 3), WHtR > 0.5 remained to be an independent risk factor of elevated CIMT (OR 1.30, 95% CI 1.01- 1.68). In three models, each1-SD increase in WHtR conveyed 1.40-fold, 1.24-fold, or and 1.25-fold higher risks of elevated CIMT, respectively (all p values < 0.05; Table 3).

**Table 3:** **Levels of WHtR in relation to the risk of elevated CIMT**

**WHtR**	**Model 1**	**p value**	**Model 2**	**p value**	**Model 3**	**p value**
≤ 0.5	1.0	—	1.0	—	1.0	—
> 0.5	1.90 (1.55-2.35)	< 0.0001	1.45 (1.14-1.83)	0.0022	1.30 (1.01-1.68)	0.0444
Each1-SD elevation	1.40 (1.29-1.53)	< 0.0001	1.24 (1.12-1.37)	< 0.0001	1.25 (1.07-1.47)	0.0064

SD, standard deviation.

Model 1: adjusted for age, sex, smoking status and alcohol drinking status.

Model 2: further adjusted for SBP, DPB, HbA1c, 2 h PG, HOMA-IR, TG, LDL-C, HDL-C and medical treatment for diabetes and hypertension based on model 1.

Model 3: further adjusted for BMI based on model 2.

### VFA and CIMT

Pearson correlation analyses revealed that age, sex, SBP, DBP, FPG, 2 h PG, HbA1c, HOMA-IR, TG, HDL-C, VFA, WHtR, and BMI were significantly associated with CIMT (p value < 0.01). Stepwise multiple linear regression analyses revealed that apart from well-recognized risk factors of CIMT such as age, sex, SBP, and HbA1c, VFA was also an independent determinant of CIMT (Table 4).

**Table 4:** **Univariate and multiple stepwise linear regression analysis of risk factors associated with CIMT in the subgroup**

	**Univariate**		**Stepwise**	
	**r**	**p value**	**β ± SE**	**p value**
Age (years)	0.1481	0.0005	0.0027 ± 0.0012	0.0273
Sex (male = 1, female = 2)	−0.2697	< 0.0001	−0.0373 ± 0.0145	0.0103
SBP (mmHg)	0.2383	< 0.0001	0.0011 ± 0.0003	< 0.0001
DBP (mmHg)	0.2397	< 0.0001	—	—
HbA1c (%)	0.2008	< 0.0001	0.4848 ± 0.1359	0.0004
FPG (mmol/L)	0.1158	0.0070	—	—
2 h PG (mmol/L)	0.1397	0.0011	—	—
HOMA-IR	0.1153	0.0072	—	—
TC (mmol/L)	0.0097	0.8218	—	—
TG (mmol/L)	0.1261	0.0033	—	—
LDL-C (mmol/L)	0.0525	0.2226	—	—
HDL-C (mmol/L)	−0.1173	0.0063	—	—
VFA (cm^2^)	0.2854	< 0.0001	0.0004 ± 0.0001	0.0014
SFA (cm^2^)	−0.0434	0.3122	—	—
BMI (kg/m^2^)	0.1879	< 0.0001	—	—

VFA, visceral fat area; SFA, subcutaneous fat area.

## Discussion

The present study provided important evidence that central fat accumulation, as indicated by increased WHtR and VFA, was associated with carotid atherosclerosis independent of general fat distribution and other risk factors in middle-aged and elderly Chinese without history of CVD.

Prospective data from the Shanghai Women’s Health Study revealed that high levels of BMI, WHR, WC, and WHtR were all associated with significantly increased risk of total, ischemic, and hemorrhagic stroke [19]. Observations from seventy-eight prospective and cross-sectional studies suggested that although WHtR, WC, and BMI were all predictors of CVD, diabetes and related disorders, WHtR and WC were probably more reliable predictors than BMI [20]. Gelber RP et al. [21] demonstrated that WHtR was statistically the best model fit and had strongest associations with CVD, compared with WC, WHR and BMI. Early detection and intervention of risk factors of CVD are the key points for prevention and delay of CVD development. CIMT is an early indicator of CVD being widely adopted [22,23] and BMI is recognized as an index of general fat distribution. A number of previous studies [24-27] have demonstrated the relationship between obesity and carotid atherosclerosis. However, studies evaluating the associations between WHtR or VFA and CIMT are limited.

In our study, subjects with central obesity alone (NW-C) exhibited higher prevalences of cardiometabolic risk factors and increased CIMT than subjects with general overweight/obesity (OW/OB-NC). Jose I Recio-Rodrigue et al. [28] reported that measures of central obesity (WHtR and WC) correlated better than BMI and body fat percentage with subclinical atherosclerosis evaluated by CIMT, independent of the presence of diabetes or hypertension. Our study also showed that WHtR was significantly associated with CIMT after adjustment for BMI and a set of cardiometabolic risk factors. A systematic review and meta-analysis confirmed that WHtR was proved to be a superior tool for discriminating obesity-related cardiometabolic risks compared with BMI [12], and that WHtR might be a more useful clinical screening tool than WC, with a weighted mean boundary value of 0.5 [20]. In addition, VFA was an independent risk factor for subclinical atherosclerosis rather than BMI and SFA after adjustment for cardiometabolic risk factors in our study. These results illustrated that central fat accumulation was more important than general fat accumulation in terms of affecting cardiometabolic and atherosclerotic risks. Lee MJ et al. [29] reported that visceral fat thickness, but not subcutaneous fat thickness, was independently associated with carotid atherosclerosis determined by CIMT in peritoneal dialysis patients. Our study found a similar association of visceral fat accumulation calculated as VFA with CIMT in a general Chinese population. Although VFA is more accurate than WHtR, the measurement of VFA is costly and complicated. WHtR is obviously more convenient in clinical practice.

Why does central fat accumulation correlate with atherosclerosis? Some investigators reported that obesity-associated systemic inflammation and oxidative stress played a key role in the initiation, propagation, and development of atherosclerosis [30]. Adipokines such as leptin and plasminogen activator inhibitor type 1 are mostly produced by visceral adipose tissue. These adipokines play important regulatory roles in a variety of biological processes, including insulin resistance and atherosclerosis [31-36]. Indulekha K et al. [37] reported that visceral fat accumulated with increasing glucose intolerance and was associated with decreased levels of adiponectin and increased levels of high sensitive C-reactive protein, tumor necrosis factor-alpha, oxidized LDL, HOMA-IR and CIMT. Nakanishi-Minami T et al. [38] reported that the duration of diabetes was an important factor in the development of coronary artery disease and visceral fat reduction might be an important approach to improving insulin resistance for patients with visceral fat accumulation. Another study showed that HOMA-IR and visceral adiposity, but not BMI, can be used to categorize morbidly obese subjects with or without metabolic and vascular impairment who are thus potentially at risk of future cardiovascular events [39]. Plasma thiobarbituric acid reactive substances, a biomarker of oxidative stress, was most strongly associated with visceral adipose tissue (VAT), which further implicates VAT as a pro-atherogenic region of adiposity. VAT is involved in the generation of systemic oxidative stress and such redox perturbations may play a causal role in the early subclinical stages of CVD [40].

The present study has several limitations. First, due to the cross-sectional study design, our findings could not prove causal relationships between central fat accumulation and elevated CIMT. Second, the subgroup with VFA measurement had a relatively small sample size. Third, participants in this study were middle-aged and elderly Chinese, limiting the generalizability of our findings to other ethnic and age groups.

## Conclusion

In conclusion, we have demonstrated that WHtR and VFA were associated with CIMT, an early marker of atherosclerosis, independent of BMI and other cardiometabolic risk factors. Given the relatively high cost and complexness of VFA measurement, WHtR could be a more convenient and appropriate measure of abdominal obesity in clinical practice.

## Abbreviations

CVD: Cardiovascular disease
WHtR: Waist-to-height ratio
WC: Waist circumference
WHR: Waist-to-hip ratio
VFA: Visceral fat area
CIMT: Carotid intima-media thickness
BMI: Body mass index
OW/OB-NC: Overweight/obesity but not central obesity group
NW-C: Normal weight but central obesity group
OW/OB-C: Overweight/obesity and central obesity group
CT: Computed tomography
ECG: Electrocardiogram
BP: Blood pressure
2 h PG: 2 h post-load glucose
SFA: Subcutaneous fat area
FPG: Fasting plasma glucose
TG: Triglycerides
TC: Total cholesterol
LDL-C: Low-density lipoprotein cholesterol
HDL-C: High-density lipoprotein cholesterol
HbA1c: Hemoglobin A1c
HOMA-IR: Homeostasis model assessment of insulin resistance
SD: Standard deviation
OR: Odds ratio
CI: Confidence interval
SBP: Systolic BP
DBP: Diastolic BP
VAT: Visceral adipose tissue

## References

[1] Eckel RH, Krauss RM, Aha nutrition committee (1998). American heart association call to action: Obesity as a major risk factor for coronary heart disease. Circulation.

[2] Evans PD, McIntyre NJ, Fluck RJ, McIntyre CW, Taal MW (2012). Anthropomorphic measurements that include central fat distribution are more closely related with key risk factors than bmi in ckd stage 3. PLoS ONE.

[3] Nissen SE, Nicholls SJ, Wolski K, Rodés-Cabau J, Cannon CP, Deanfield JE, Després JP, Kastelein JJ, Steinhubl SR, Kapadia S, Yasin M, Ruzyllo W, Gaudin C, Job B, Hu B, Bhatt DL, Lincoff AM, Tuzcu EM, STRADIVARIUS Investigators (2008). Effect of rimonabant on progression of atherosclerosis in patients with abdominal obesity and coronary artery disease: The stradivarius randomized controlled trial. JAMA.

[4] Goodpaster BH, Krishnaswami S, Resnick H, Kelley DE, Haggerty C, Harris TB, Schwartz AV, Kritchevsky S, Newman AB (2003). Association between regional adipose tissue distribution and both type 2 diabetes and impaired glucose tolerance in elderly men and women. Diabetes Care.

[5] Tulloch-Reid MK, Hanson RL, Sebring NG, Reynolds JC, Premkumar A, Genovese DJ, Sumner AE (2004). Both subcutaneous and visceral adipose tissue correlate highly with insulin resistance in african americans. Obes Res.

[6] Wagenknecht LE, Langefeld CD, Scherzinger AL, Norris JM, Haffner SM, Saad MF, Bergman RN (2003). Insulin sensitivity, insulin secretion, and abdominal fat: The insulin resistance atherosclerosis study (iras) family study. Diabetes.

[7] Boyko EJ, Fujimoto WY, Leonetti DL, Newell-Morris L (2000). Visceral adiposity and risk of type 2 diabetes: A prospective study among japanese americans. Diabetes Care.

[8] Kanaya AM, Harris T, Goodpaster BH, Tylavsky F, Cummings SR, Health, Aging, and Body Composition (ABC) Study (2004). Adipocytokines attenuate the association between visceral adiposity and diabetes in older adults. Diabetes Care.

[9] Lemieux I, Pascot A, Couillard C, Lamarche B, Tchernof A, Alméras N, Bergeron J, Gaudet D, Tremblay G, Prud’homme D, Nadeau A, Després JP (2000). Hypertriglyceridemic waist: A marker of the atherogenic metabolic triad (hyperinsulinemia; hyperapolipoprotein b; small, dense ldl) in men?. Circulation.

[10] Dahlén EM, Bjarnegård N, Länne T, Nystrom FH, Ostgren CJ (2013). Sagittal abdominal diameter is a more independent measure compared with waist circumference to predict arterial stiffness in subjects with type 2 diabetes - a prospective observational cohort study. Cardiovasc Diabetol.

[11] Ashwell M, Hsieh SD (2005). Six reasons why the waist-to-height ratio is a rapid and effective global indicator for health risks of obesity and how its use could simplify the international public health message on obesity. Int J Food Sci Nutr.

[12] Ashwell M, Gunn P, Gibson S (2012). Waist-to-height ratio is a better screening tool than waist circumference and BMI for adult cardiometabolic risk factors: systematic review and meta-analysis. Obes Rev.

[13] Mohammadreza B, Farzad H, Davoud K, Fereidoun Prof AF (2012). Prognostic significance of the Complex “Visceral Adiposity Index” vs. simple anthropometric measures: Tehran lipid and glucose study. Cardiovasc Diabetol.

[14] Grobbee DE, Bots ML (1994). Carotid artery intima-media thickness as an indicator of generalized atherosclerosis. J Intern Med.

[15] Dahlen EM, Lanne T, Engvall J, Lindström T, Grodzinsky E, Nystrom FH, Ostgren CJ (2009). Carotid intima-media thickness and apolipoprotein b/apolipoprotein a-i ratio in middle-aged patients with type 2 diabetes. Diabet Med.

[16] Ashwell M, Gibson S (2009). Waist to height ratio is a simple and effective obesity screening tool for cardiovascular risk factors: Analysis of data from the british national diet and nutrition survey of adults aged 19–64 years. Obes Facts.

[17] Gordon-Larsen P, Adair LS, Meigs JB, Mayer-Davis E, Herring A, Yan SK, Zhang B, Du S, Popkin BM (2013). Discordant risk: Overweight and cardiometabolic risk in chinese adults gordon-larsen: Overweight and cardiometabolic risk in china. Obesity (Silver Spring).

[18] WHO Expert Consultation (2004). Appropriate body-mass index for asian populations and its implications for policy and intervention strategies. Lancet.

[19] Zhang X, Shu XO, Gao YT, Yang G, Li H, Zheng W: Zhang X, Shu XO, Gao YT, Yang G, Li H, Zheng W (2009). General and Abdominal Adiposity and Risk of Stroke in Chinese Women. Stroke.

[20] Browning LM, Hsieh SD, Ashwell M (2010). A systematic review of waist-to-height ratio as a screening tool for the prediction of cardiovascular disease and diabetes: 0 · 5 could be a suitable global boundary value. Nutr Res Rev.

[21] Gelber RP, Gaziano JM, Orav EJ, Manson JE, Buring JE, Kurth T (2008). Measures of obesity and cardiovascular risk among men and women. J Am Coll Cardiol.

[22] Bots ML, Baldassarre D, Simon A, de Groot E, O’Leary DH, Riley W, Kastelein JJ, Grobbee DE (2007). Carotid intima-media thickness and coronary atherosclerosis: Weak or strong relations?. Eur Heart J.

[23] Williams K, Sniderman AD, Sattar N, D’Agostino R, Wagenknecht LE, Haffner SM (2003). Comparison of the associations of apolipoprotein b and low-density lipoprotein cholesterol with other cardiovascular risk factors in the insulin resistance atherosclerosis study (iras). Circulation.

[24] Kotsis VT, Stabouli SV, Papamichael CM, Zakopoulos NA (2006). Impact of obesity in intima media thickness of carotid arteries. Obesity (Silver Spring).

[25] Bigazzi R, Bianchi S, Batini V, Guzzo D, Campese VM (2006). Metabolic risk factors and markers of cardiovascular and renal damage in overweight subjects. Am J Hypertens.

[26] Karason K, Wikstrand J, Sjostrom L, Wendelhag I (1999). Weight loss and progression of early atherosclerosis in the carotid artery: A four-year controlled study of obese subjects. Int J Obes Relat Metab Disord.

[27] Park JS, Cho MH, Ahn CW, Kim KR, Huh KB (2012). The association of insulin resistance and carotid atherosclerosis with thigh and calf circumference in patients with type 2 diabetes. Cardiovasc Diabetol.

[28] Recio-Rodriguez JI, Gomez-Marcos MA, Patino-Alonso MC, Agudo-Conde C, Rodriguez-Sanchez E, Garcia-Ortiz L, Vasorisk group (2012). Abdominal obesity vs general obesity for identifying arterial stiffness, subclinical atherosclerosis and wave reflection in healthy, diabetics and hypertensive. BMC Cardiovasc Disord.

[29] Lee MJ, Shin DH, Kim SJ, Oh HJ, Yoo DE, Kim JK, Park JT, Han SH, Kang SW, Choi KH, Yoo TH (2012). Visceral fat thickness is associated with carotid atherosclerosis in peritoneal dialysis patients. Obesity (Silver Spring).

[30] Hulsmans M, De Keyzer D, Holvoet P (2011). Micrornas regulating oxidative stress and inflammation in relation to obesity and atherosclerosis. FASEB J.

[31] Reyes M, Gahagan S, Diaz E, Blanco E, Leiva L, Lera L, Burrows R (2011). Relationship of adiposity and insulin resistance mediated by inflammation in a group of overweight and obese chilean adolescents. Nutr J.

[32] Mathieu P, Lemieux I, Despres JP (2010). Obesity, inflammation, and cardiovascular risk. Clin Pharmacol Ther.

[33] Filkova M, Haluzik M, Gay S, Senolt L (2009). The role of resistin as a regulator of inflammation: Implications for various human pathologies. Clin Immunol.

[34] Beltowski J (2006). Leptin and atherosclerosis. Atherosclerosis.

[35] Oliveira MA, Fagundes RL, Moreira EA, Trindade EB, Carvalho TD (2010). Relation between anthropometric indicators and risk factors for cardiovascular disease. Arq Bras Cardiol.

[36] Costa GB, Horta N, Resende ZF, Souza G, Barreto LM, Correia LH, Nascimento TA, Rios CB, Barreto-Filho JA, Lopes HF (2009). Body mass index has a good correlation with proatherosclerotic profile in children and adolescents. Arq Bras Cardiol.

[37] Indulekha K, Anjana RM, Surendar J, Mohan V (2011). Association of visceral and subcutaneous fat with glucose intolerance, insulin resistance, adipocytokines and inflammatory markers in asian indians (cures-113). Clin Biochem.

[38] Nakanishi-Minami T, Kishida K, Nakagawa Y, Nakatsuji H, Kuroda Y, Okauchi Y, Yamasaki K, Nojima Y, Tsujii K, Kumada M, Tachibana K, Nakamura T, Sumitsuji S, Funahashi T, Shimomura I (2012). Carotid intima-media thickness, but not visceral fat area or adiponectin, correlates with intracoronary stenosis detected by multislice computed tomography in people with type 2 diabetes and hypertension. Diabetes Res Clin Pract.

[39] Lupattelli G, De Vuono S, Boni M, Helou R, Raffaele Mannarino M, Rita Roscini A, Alaeddin A, Pirro M, Vaudo G (2013). Insulin resistance and not BMI is the major determinant of early vascular impairment in patients with morbid obesity. J Atheroscler Thromb.

[40] Lear SA, Sarna LK, Siow TJ, Mancini GB, Siow YL, O K (2012). Oxidative stress is associated with visceral adipose tissue and subclinical atherosclerosis in a healthy multi-ethnic population. Appl Physiol Nutr Metab.

